# Endoscopic sinus surgery in adult patients with chronic rhinosinusitis with nasal polyps (PolypESS): study protocol for a randomised controlled trial

**DOI:** 10.1186/s13063-016-1728-z

**Published:** 2017-01-23

**Authors:** Evelijn S. Lourijsen, Corianne A. J. M. de Borgie, Marleen Vleming, Wytske J. Fokkens

**Affiliations:** 1 Department of Otorhinolaryngology, Academic Medical Centre, Meibergdreef 9, 1105 SZ Amsterdam, The Netherlands; 2 Clinical Research Unit, Academic Medical Centre, Meibergdreef 9, 1105 SZ Amsterdam, The Netherlands; 3 Department of Otorhinolaryngology, Flevo Hospital, Hospitaalweg 1, 1315 RA Almere, The Netherlands

**Keywords:** Sinusitis, Nasal polyps, Chronic disease, Surgery, Endoscopy, Endoscopic surgical procedure, Drug therapy, Outcome, Quality of life, Cost-effectiveness analysis

## Abstract

**Background:**

Chronic rhinosinusitis with nasal polyps is a chronic disease frequently seen in otorhinolaryngological practice. Along with its chronic disease burden it creates high societal costs. Therapy consists of long-term use of medication and, if insufficient, endoscopic sinus surgery. No consensus exists on the right timing and extent of disease that warrants surgery. Furthermore, there is lack of clinical knowledge about the benefit of surgery over medication only. The current trial evaluates the clinical effectiveness and cost-effectiveness of endoscopic sinus surgery in addition to drug treatment versus medication exclusively in the adult patient group with nasal polyps.

**Methods:**

A prospective, multicentre, superiority, randomised controlled (PolypESS) trial in 238 patients aged 18 years or older selected for primary or revision endoscopic sinus surgery by the otorhinolaryngologist was designed. Patients will be randomised to either endoscopic sinus surgery in addition to medication or medical therapy only. Relevant data will be collected prior to randomisation, at baseline and 3, 6, 12, 18 and 24 months after start of treatment. Complete follow-up will be 24 months. Primary outcome is disease-specific Health-related Quality of Life quantified by the SNOT-22 after 12-month follow-up. Secondary outcomes are generic Health-related Quality of Life, cost-effectiveness, objective signs of disease and adverse effects of treatment. Subgroup analyses will be performed to verify whether treatment effects differ among patient phenotypes.

**Discussion:**

The PolypESS trial will investigate tailored care in adult patients with chronic rhinosinusitis with nasal polyps and will result in improved clinical pathways to help to determine in which circumstances to perform surgery.

**Trial registration:**

Dutch Trial Register, NTR4978. Registered on 27 November 2014.

**Electronic supplementary material:**

The online version of this article (doi:10.1186/s13063-016-1728-z) contains supplementary material, which is available to authorized users.

## Background

Chronic rhinosinusitis (CRS) can manifest as a disease with nasal polyps (CRSwNP) or without nasal polyps (CRSsNP). The prevalence of both forms of CRS in Europe is around 11% [1]. CRSwNP is the more serious form of CRS and is associated with a prevalence of 1–4% [2]. Patients with CRS experience a significant impact on most aspects of Health-related Quality of Life (HRQOL) and investigation has shown this to exceed the impact on HRQOL of patients with chronic heart failure, diabetes and chronic back pain [3, 4]. The high prevalence and significant negative impact on most aspects of HRQOL burdens the diagnostic process and treatment with high medical resource usage and high societal costs [1].

(Inter-)national clinical CRS guidelines advise starting drug treatment for at least 1 month before considering surgery; however, there is no guideline that advises or specifies conditions that warrant surgery [3, 5, 6]. Currently, patients failing drug treatment are offered a more intensive drug regimen or endoscopic sinus surgery (ESS) in addition to drug treatment. In The Netherlands most otorhinolaryngologists prefer surgery, though practice variance is high with regard to the timing and rationale of ESS. These differences lead to inefficient health care practice. Also, if ESS is not proven to be (cost-)effective, risks are generated in exposing patients to ineffective treatment.

A national audit in the UK demonstrated that 69% of ESS is performed for CRSwNP [7]. Corresponding data in The Netherlands are lacking, but may be expected to be similar. A recent Chronic Rhinosinusitis Epidemiology Study (CRES) performed in the UK demonstrated that from all respondents with CRSwNP (*N* = 651) 57% underwent previous sinonasal surgery and 20% underwent multiple surgeries [8]. This high burden of primary and revision surgery remains unclear in aetiology, but highlights the need for more research concerning endotyping and phenotyping patients with CRSwNP as well as more research concerning aspects of surgery itself.

A recent analysis of the National Comparative Audit of Surgery for Nasal Polyposis and Chronic Rhinosinusitis depicted that almost 40% of patients undergoing ESS suffered more than 5 years from their symptoms related to CRS [9]. Hopkins et al. specifically looked at the timing of ESS and its influence on symptoms. In the National Comparative Audit of Surgery for Nasal Polyposis and Chronic Rhinosinusitis [7] the effect of patient time to surgery on symptomatic outcomes was evaluated and it was found that patients treated at an early stage in the course of disease (i.e. within 12 months after first diagnosis of CRS) experience more improvement in symptoms after surgical intervention compared to patients treated after a longer burden of CRS (i.e. after 5 years from first diagnosis of CRS) [9]. On top of this finding, the cohort of patients treated after a longer period of CRS had greater CRS-related health care needs postoperatively, consisting of medical visits and prescriptions per patient per year [10]. This available data raises more questions about the right timing of ESS.

The systematic review of the Cochrane Review Group on ESS for CRS (2006) concluded, with the limited evidence available, that ESS has not demonstrated an additional benefit in comparison to drug treatment. The need for more randomised trials was highlighted in the review [11].

A recent non-randomised, multicentre cohort study by Smith et al. looked at the differences in HRQOL in a CRS patient group that self-selected ESS versus a patient group that self-selected drug treatment. They demonstrated a significantly better improvement in HRQOL in addition to less use of systemic medication usage after ESS in comparison to on-going medication until 6-month follow-up [12]. They also found a better HRQOL in patients that self-selected ESS at 1-year follow-up [13].

Scientific evidence for the effectiveness and the severity of disease that warrants ESS, ideally retrieved from a well-designed randomised controlled trial, is missing. The aim of the present trial is to investigate in a randomised fashion whether two regularly applied treatment strategies used in adult patients with CRSwNP, ESS in addition to drug treatment or drug treatment only, differ in generic and disease-specific HRQOL and to establish the presumed superiority of ESS. A comparison with respect to cost-effectiveness will also be made.

## Methods

### Study objectives

The primary aim of the PolypESS trial is to assess the effectiveness of ESS in addition to drug treatment as compared to drug treatment alone in adults with CRSwNP in terms of improving patients’ HRQOL, measured by the Sinonasal Outcome Test 22 (SNOT-22) at 12-month follow-up. Key secondary aims of the trial are evaluation of the effectiveness of ESS in addition to drug treatment as compared to drug treatment alone in the short (3–6 months) and long (12–24 months) term, in terms of generic HRQOL, objective signs of disease and adverse effects of treatment. This trial will also evaluate which patient phenotypes within CRSwNP benefit from ESS in addition to drug treatment as compared to drug treatment alone. Furthermore, the relation between health care resource use and patient costs and effects of ESS will be determined from a societal point of view. It is hypothesised that a more tailored approach for patients with CRSwNP will be associated with lower medical and indirect costs (health care utilisation and productivity loss).

### Study design

**Fig. 1:**
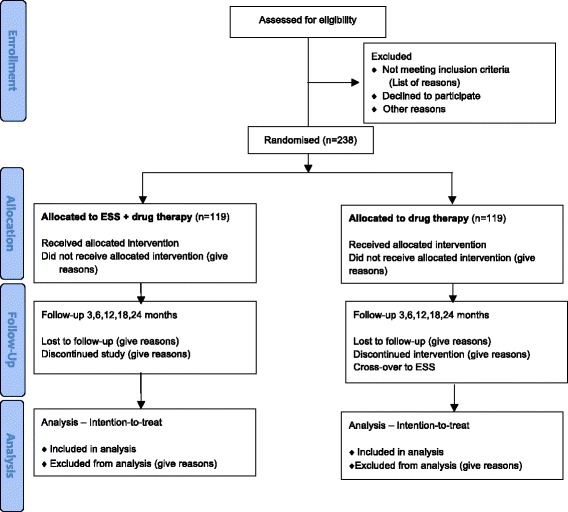
CONsolidated Standards of Reporting Trials (CONSORT) flow diagram of PolypESS study (ESS, endoscopic sinus surgery)

The PolypESS is an investigator-initiated, prospective, open, national, multicentre randomised controlled trial investigating the (cost-)effectiveness of ESS in patients suffering from CRSwNP. Suitable patients will be randomised into two treatment groups. In the first group patients will undergo ESS in addition to medication, in the second group patients will receive an intensified drug treatment only. Total follow-up is 24 months for all included consecutive patients. Otorhinolaryngologists in the participating centres are asked to recruit patients. Any patient who meets the inclusion criteria (described in detail below in ‘Study population’ section) will be informed about the trial and asked to participate. The coordinating trial centre (Academic Medical Centre, Amsterdam, The Netherlands) will contact patients who have expressed interest in the trial. The study team member will provide detailed written and oral information about the trial and answer any questions. If patients agree to participate, inclusion and exclusion criteria will be checked and a baseline visit will be scheduled. Potential participants have sufficient time before they give their final consent to participate in the trial. If patients decline participation, known clinical data on disease-specific HRQOL and objective signs of disease are used to evaluate whether the findings of the consecutive sampling are generalisable to the target population. The trial reporting is according to the CONsolidated Standards of Reporting Trials (CONSORT) guidelines and the CONSORT extension for nonpharmacological interventions (Fig. 1) [14, 15]. The Standard Protocol Items: Recommendations for Interventional Trials (SPIRIT) 2013 Checklist: ‘Recommended items to address in a clinical trial protocol and related documents’, can be found in Additional file 1. The SPIRIT participant schedule is shown in Table 1.

**Table 1:** Standard Protocol Items: Recommendations for Interventional Trials (SPIRIT) schedule patient enrolment, interventions and assessments

	Visit -2	Visit -1	Visit 0	Visit 1	Visit 2	Visit 3	Telephone consult	Visit 4
Timepoints	<10 wks	<6 wks		3 months	6 months	12 months	18 months	24 months
ENT-surgeons asks patient to participate	x							
Informed Consent		x						
Demographic Data		x						
Medical History including earlier sinus surgery		x						
Inclusion Criteria		x						
Exclusion criteria		x						
Randomisation		x						
ESS or intensify medical treatment based on randomisation			x					
Vital Signs and bodyweight		x						
Symptoms		x		x	x	x	x	x
Disease specific HRQOL and symptoms (SNOT-22)		x		x	x	x	x	x
Generic HRQOL (EQ-5D-5L)		x		x	x	x	x	x
Endoscopic assessment of the nose		x		x	x	x		x
Olfactory function (Sniffin’ Sticks)		x		x	x	x		x
Nasal obstruction (PNIF)		x		x	x	x		x
Daily records cards (DRC)		x		x	x	x	x	x
CRS disease control		x		x	x	x		x
Asthma control		x		x	x	x	x	x
Healthcare resource use				x	x	x	x	x
Adverse effects				x	x	x	x	x
CT scan (Lund-Mackay score)		x						
Surgical report			x					
Laboratory tests, pregnancy test		x						
Skin Prick test (if done in the last year, results are recorded)		x						

*Wks* weeks, *HRQOL* health-related quality of life, *SNOT-22* sinonasal outcome test 22, *EQ-5D-5L* EuroQol-5D-5L questionnaire, *PNIF* peak nasal inspiratory flow

### Setting

The trial is performed in 15 hospitals, three university-affiliated hospitals (The Academic Medical Centre, Amsterdam; Erasmus Medical Centre, Rotterdam; VU Medical Centre, Amsterdam) and teaching hospitals (Amstelland Hospital, Amstelveen; Alrijne Hospital, Leiderdorp and Alphen aan den Rijn; BovenIJ Hospital, Amsterdam; Deventer Hospital, Deventer; Flevo Hospital, Almere; Haga Hospital, Den Haag; Onze Lieve Vrouwe Gasthuis location East, Amsterdam; Onze Lieve Vrouwe Gasthuis location West, Amsterdam; Spaarne Hospital, Hoofddorp; Spaarne Hospital, Haarlem; Tergooi Hospital, Hilversum and Blaricum; and Westfries Gasthuis, Hoorn) in The Netherlands. The same medical researcher and research nurses perform all trial assessments in the participating centres, apart from nasal endoscopy. Nasal endoscopy is performed by the local ENT surgeon as part of standard care.

### Study population

Adult patients (18 years of age or older) with bilateral CRSwNP who have been selected by their ENT surgeon as candidates for primary or revision ESS are eligible for participation. If patients are excluded from participation, reason(s) for exclusion are registered. Exclusion criteria include the presence of systemic diseases affecting the nose (e.g. Wegener’s granulomatosis, sarcoidosis, primary ciliary dyskinesia, cystic fibrosis), antrochoanal polyps, malignant polyps, inverted papilloma, sinonasal tumours, absolute need for surgical therapy, contraindications for surgical therapy, need for radical surgery (Draf III, Denker surgery, medial maxillectomy), polypectomy without ethmoidectomy, continuous use of systemic corticosteroids for diseases other than CRSwNP, continuous medication for other diseases influencing CRSwNP (e.g. other immunosuppressive drugs), pregnancy at enrolment, mental or systemic illnesses preventing adequate participation in the trial and any other scheduled surgical intervention preventing adequate participation in the trial. Furthermore, potential participants are not allowed to have used any systemic corticosteroids in the previous 4 weeks before enrolment and they should not have suffered from an acute upper or lower respiratory tract infection at the time of enrolment or during the previous 2 weeks.

### Patient enrolment

Selection of patients follows a two-stage procedure. Consecutive patients are screened for eligibility by a recruiting local otorhinolaryngologist in the outpatient department. Additionally a telephone interview is scheduled by the medical researcher in the Academic Medical Centre during which eligibility will be reassessed. Patients will be enrolled by the medical researcher during a clinic visit in the concerning hospital. Patients meeting all inclusion criteria and no exclusion criteria can be included and will be randomly assigned, after informed consent is given.

### Baseline measurements

On the day of enrolment, patients will undergo the same HRQOL and objective evaluation, including the SNOT-22, the EuroQol-5D-5L questionnaire (EQ-5D-5L, the EuroQol Group) and nasal endoscopy, that will be used after treatment (see “Outcomes” section). Clinical data is collected by the enrolling medical researcher and focuses on the (severity of) patient-reported symptoms, previous clinical examinations, previous sinonasal surgery, previous conservative treatment and complete medical history. Demographic variables include age, gender, ethnicity, marital status, family situation and highest level of education. Additional study information includes the presence or absence of asthma, acetylsalicylic acid (ASA) intolerance, allergy, occupational exposure, smoking habits and alcohol consumption. Objective measurements recorded are baseline height, weight, blood pressure, resting heart rate, total IgE, serum eosinophil level and computed tomography (CT) scores using the Lund-Mackay scoring system. This system uses a 0–1–2 score dependent upon absent, partial or complete opacification, respectively, of each individual sinus and the ostiomeatal complex, contributing to a maximum score of 12 per side. The total score of the two sides can reach a maximal 24 points [16]. A urine pregnancy test will be performed in female patients with childbearing potential if in doubt of pregnancy.

### Skin Prick Test

To assess allergic sensitisation, the Global Allergy and Asthma European Network’s (GA^2^LEN) standardised method of the Skin Prick Test (SPT) is used [17]. Patients are instructed to stop taking antihistamine medication 5 days before the SPT. A positive reaction to the SPT is defined as a skin reaction larger than 3 mm for one or more of the tested allergens (at least tree, grass, Dermatophagoides pteronyssinus, cat, dog, moulds) and no reaction to the negative control.

### Interventions

Patients will be assigned to a surgical strategy (ESS in addition to drug treatment) or a drug treatment strategy exclusively. Clinicians and patients will not be blinded to the treatment arm of the study. Those assigned to surgery will be offered ESS within 6 weeks of randomisation. Those assigned to drug treatment will be seen by the otorhinolaryngologist within 6 weeks of randomisation to define the need for additional medication. As this is a pragmatic trial, ESS refers to the surgery performed regularly by otorhinolaryngologists in The Netherlands. The extent of surgery is tailored to the extent of the disease. Drug treatment comprises any usual care medication.

### Outcomes

#### Primary study outcome

The effectiveness of both interventions is evaluated by the SNOT-22 after 12 months of follow-up. The SNOT-22 is a patient-reported measure of outcome (PROM) consisting of 22 individual custom-designed questions for use in CRS with or without nasal polyposis. The SNOT-22 covers a broad range of disease-specific HRQOL topics including physical complaints, functional limitations and emotional consequences. This questionnaire has shown to be reliable and valid in clinical practice to assess the impact of CRS on a patient’s disease-specific HRQOL and to measure treatment-related changes [18].

#### Secondary study outcomes

Clinical outcome data will be collected at 3, 6, 12, 18 and 24 months after start of treatment. All measurements are performed by an adequately trained medical researcher and research nurses. All measurements are carried out according to protocol procedures and defined standard operating procedures.

#### HRQOL measurements

To assess generic HRQOL the EQ-5D-5L is administered [19]. The questionnaire comprises five domains/questions: mobility, self-care, usual activities, pain or discomfort and anxiety or depression. An EQ-5D-5L index can be calculated and quantifies a participant’s health status on a scale ranging from 0 (very bad health) to 1 (perfect health). Patients are also instructed to rate their overall generic HRQOL using a Visual Analogue Scale (EuroQol-5D VAS) ranging from 0 (very bad health) to 100 (perfect health). In this study the validated Dutch translation is used.

#### Symptoms

Total clinical symptoms, symptoms of rhinorrhoea, facial pain/headache, loss of smell and nasal blockage are measured with a Visual Analogue Scale (VAS) ranging from 0 (no problem) to 10 (worst imaginable problem).

#### Olfactory function

The ‘Sniffin’ Sticks Identification Test is used to assess olfactory performance. These twelve sticks are odour-dispensing devices that resemble felt-tipped pens and are held under the participant’s nose for 3–4 s. The participant must make a forced choice from a list of four options as to the nature of the odour. The score corresponds to the amount of correct answers.

#### Nasal obstruction

The peak nasal inspiratory flow (PNIF) method is used to quantify nasal obstruction. A portable Youlten Peak Flow Meter (Clement Clarke International) is used. After applying a ventilation mask to firmly cover the nose and the mouth, participants are instructed to inhale as strongly as possible through the nose with the mouth closed. Three maximal inspirations are performed and the highest value (L/min) is used for analysis.

#### Endoscopic nasal assessment

Three different nasendoscopic measurements are used:

1. The Meltzer Clinical Scoring System is a 0–4 polyp grading system (0 = no polyps, 1 = polyps confined to the middle meatus, 2 = multiple polyps occupying the middle meatus, 3 = polyps extending beyond middle meatus, 4 = polyps completely obstructing the nasal cavity)
2. The Modified Lund-Kennedy Endoscopy Score is a 0–2 scoring system in which the endoscopic appearances of both nasal fossae are rated for polyps, oedema and discharge (*polyps*: 0 = no polyps, 1 = polyps confined to the middle meatus, 2 = polyps beyond the middle meatus; *oedema*: 0 = no oedema, 1 = mild oedema, 2 = severe oedema; *discharge*: 0 = none, 1 = clear and thin, 2 = thick and eosinophilic) [20]
3. The Modified Lund-Mackay Postoperative Endoscopy Score (MLMES) applies to all participants who previously underwent sinus surgery. The endoscopic appearances of all ten cavities (left and right maxillary, ethmoid, sphenoid and frontal sinuses and olfactory fossa) are quantified for *mucosal inflammation* (0–6: 0 = normal mucosa, 1 = mild oedematous mucosa with patent cavity, 2 = severely oedematous mucosa with compromised cavity, 3 = mild polypoid mucosa with patent cavity, 4 = severe polypoid mucosa with compromised cavity, 5 = polyp confined within cavity, 6 = polyp extending beyond cavity), *mucus* (0–2: 0 = none, 1 = clear and thin, 2 = thick and eosinophilic) and *purulent discharge* (0–2: 0 = absent, 2 = present). This system produces a score of 0–100. Draf III cavities are scored as two frontal sinuses separately. Nonpneumatised sinuses and nondiseased sinuses that have not undergone surgery are scored as 0. The olfactory fossa is evaluated by assessing the cleft between the nasal septum and the middle turbinate anteriorly and the superior turbinate posteriorly [21].

#### CRS disease control

Disease control will be evaluated as suggested by the European Position Paper on Chronic Rhinosinusitis (EPOS 2012) (see Additional file 2). Nasal blockage, rhinorrhoea/postnasal drip, facial pain/headache, olfactory function, sleep disturbance or fatigue, nasendoscopy and systemic medication needed to control disease are evaluated. Each characteristic is rated as currently controlled or partly controlled, contributing to a general conclusion of CRS being controlled, partly controlled or uncontrolled at the time of assessment [3].

#### Asthma control

As asthma is a common comorbid condition in patients with CRSwNP, the Asthma Control Test (2002 TM QualityMetric Incorporated) is used in the subpopulation of patients with asthma. This validated appraisement contains five individual questions to assess asthma disease control.

#### Diaries

Participants are instructed to complete a diary 2 weeks before a follow-up visit until 2 weeks after a follow-up visit. Daily nasal symptoms and medication compliance will be recorded. The diary is also suitable to record symptomatic exacerbations, other medical problems and adverse effects or events. The nasal symptom scores, used to evaluate efficacy as a measure of compliance, will be calculated from the daily subject-rated scores of four nasal symptoms: headache/facial pain, rhinorrhoea, nasal congestion and loss of smell. Severity of symptoms is scored on a 0 to 3 scale; 0 = none (symptom is not present), 1 = mild (sign/symptom is clearly present but minimal awareness; easily tolerated), 2 = moderate (definite awareness of sign/symptom that is bothersome but tolerable), 3 = severe (sign/symptom is hard to tolerate; causes interference with activities of daily living and/or sleeping). Subjects will be instructed to score and document their symptoms every 24 h in a reflective manner using the (electronic) diary.

#### Exacerbations and adverse effects

Medical files and patient diaries are used to record any unwanted side effects and readmissions during the study period. Participants are actively queried every follow-up visit as to whether they experienced any complications or adverse effects.

#### Health care resource use and costs

Resource use and costs of health care utilisation, out-of-pocket expenses and lost productivity are retrieved from hospital databases, financial reports, medical files, patient diaries and a modified version of the Erasmus iMTA ‘Productivity Cost Questionnaire’ and modified iMTA ‘Medical Consumption Questionnaire’ [22, 23].

### Data collection, management and storage

Source documents are a custom-designed paper Case Report Form and patient medical files. In addition, electronic questionnaires are used whenever possible (Limesurvey®). All VAS questionnaires are carried out on paper. The electronic diary is compatible across all browsers (https://kno-polypess.minddistrict.nl), smartphones and tablet devices (Minddistrict® application). Participants receive a personal username and create a password. If electronic device utilisation is not feasible, paper diaries are administered.

Clinical data will be stored in a custom-designed, password-protected study database (OpenClinica® software). Paper Case Report Forms, paper questionnaires and signed informed consents are stored in locked cabinets. A Data Monitoring Committee is allowed to access the collected clinical data mother file, wherein no identifiable patient data is stored; unique patient identification codes are used instead.

### Sample size

PolypESS is a superiority trial in which disease-specific HRQOL, measured with the SNOT-22 at 12-month follow-up, is the primary outcome of interest. The sample size calculation is build on the literature-based assumption that the minimal clinically important difference (MCID) for SNOT-22 is 8.9 points (SD 20.0) [18]. Using a 5% significance level and a power of 90% yields a sample size of 238 patients, which includes a 10% anticipated loss to follow-up.

### Randomisation

A randomisation sequence is generated using block sizes of 6 stratified by study centre. A central, password-protected, consistently available automated randomisation system (ALEA® software, Trans European Network for clinical trial services (TenALEA) consortium, Amsterdam, The Netherlands) has been developed by the independent Clinical Research Unit in the Academic Medical Centre, Amsterdam, The Netherlands. Due to the nature of both interventions, blinding is not possible.

### Statistical methods

#### Primary data analysis

Results will be based on the intention-to-treat method. In addition, per-protocol analysis, including only patients who adhered completely to the clinical trial instructions and treatment specified in the protocol, will also be performed to check the robustness of results. Continuous normally distributed variables will be expressed by their mean and standard deviation or, when not normally distributed, as medians and their interquartile ranges. Categorical variables will be expressed as counts (*n*) and percentages (%). Effects on HRQOL, nasal endoscopy and symptom score will be calculated as mean differences with 95% confidence intervals. HRQOL, short- and long-term effects will be additionally evaluated at 3, 6, 12, 18 and 24 months’ follow-up, respectively. It is expected that randomisation will balance patients’ baseline characteristics. However, if imbalances occur between groups that are related to possible effect modification, subgroup analyses will be performed according to the indication for surgery.

A further detailed statistical analysis plan will be developed and reported by the chief investigators prior to the database being locked at the end of follow-up for final analysis.

#### Cost-effectiveness analysis (CEA)

##### General considerations

Alongside the randomised clinical trial an economic study will be performed. The economic evaluation will be set up as a cost-effectiveness analysis (CEA). The CEA focuses on the possible gained benefits of ESS in addition to drug treatment versus drug treatment alone and the related health care costs. The economic evaluation will be performed from a societal point of view.

##### Patient outcomes

The SNOT-22 will be measured to evaluate impact of both treatments. This will be used as endpoint in the economic evaluation. The cost-effectiveness of the interventions will be compared by assessing cost per Quality-adjusted Life Year (QALY), calculated from the health utility scores obtained with the *EQ-5D-5L*.

##### Cost analysis

Costs will be primarily assessed by the intervention study (and not the additional costs of underlying comorbid diseases). The time horizon of this cost analysis will be limited to 12-month follow-up. With this time horizon no discounting of costs and effects will be performed. The societal perspective captures the value of all resources used. Costs associated with treatment from a long-term perspective will be estimated and incorporated in a scenario analysis. Subgroup analyses will be done. Overall costs will be compared across the treatment groups and, where relevant, differences will be calculated, inclusive of 95% confidence intervals. Incremental cost-effectiveness ratios (iCERs) will be calculated.

##### Measurements

The prospective cost evaluation will primarily focus on health care utilisation (direct medical costs), travel expenses (direct nonmedical costs) and lost productivity (indirect costs) due to absence from work or decreased performance at work (productivity loss). The direct medical costs include the costs of all procedures associated with both treatment strategies (e.g. doctor’s visits, medication, hospital admissions and surgical interventions, sinus CT, endoscopy and exacerbations).

Out-of-pocket expenses include additional over-the-counter drugs and travel costs. Additional costs as a result of comorbid conditions (e.g. asthma) will be excluded.

In the base case analysis, indirect costs (based on lost productivity) will be calculated using the friction cost method. Productivity losses will be estimated based on data concerning absence from work. Health service resource use and costs of both treatment strategies will be measured from a health service and societal perspective. Protocol-driven costs will be excluded.

##### Unit costs

Costs are defined as the volumes of used resources multiplied by calculated unit prices. For the valuation of health care utilisation, standard prices published in the Dutch costing guidelines and market prices will be used [24]. Standard guideline prices will be used for all diagnostic interventions, hospital admissions, post-operative care, outpatient visits and travelling.

##### Statistical analysis

As most volumes of resource utilisation follow a skewed distribution, differences between the two groups will be statistically evaluated with bias-corrected bootstrap analysis. An iCER will be calculated, with the observed HRQOL as effect parameter.

The economic analysis will be expanded with a scenario-analysis to extrapolate the consequences of implementation and actual performance of the screening strategy in the targeted population. In sensitivity analysis the validity of the developed scenarios is evaluated. Uncertainty will be addressed by means of bootstrapping.

#### Budget impact analysis (BIA)

##### General considerations

In addition to the assessment of cost-effectiveness, a budget impact analysis (BIA) will be performed to determine the potential financial impact of more tailored treatment for patients with CRSwNP on national total health care costs in the future. The analysis will be performed according to the ISPOR Task Force guidelines [25]. The BIA will be conducted from the viewpoint of the publicly funded health and social care system. No discounting will be applied, tariffs and prices will be held constant over the years.

### Cost analysis

The BIA will be based on clinical data that reflect the size and characteristics of the population, the current treatment mix, the effectiveness of ESS, and the resource use and costs for ESS (surgery, post-operative care, exacerbations and reinterventions).

### Monitoring, safety and reporting of adverse events

An independent Good Clinical Practice (GCP)-certified Monitoring Committee has been established. Members of the independent Clinical Research Unit of the Academic Medical Centre will perform monitoring and it will be conducted according to International Conference on Harmonisation (ICH)-GCP guidelines. A detailed study-specific monitor plan (version 3, 1 August 2016) has been formulated. The monitor plan is designed to verify that the rights and wellbeing of the participants is protected, that the reported trial data are accurate, complete and verifiable from the source documents and that the conduct of the trial is in compliance with the currently approved protocol, GCP and applicable regulatory requirements. Every participating site will be physically visited at least once during the study period. All other monitoring activities will be centralised to detect sources of data irregularities, by exploring the clinical trial database. The monitoring plan will be updated and revised as needed.

The risk of the current trial is estimated to be low. Patients will only be asked to participate if there is an indication for surgery as decided by their otorhinolaryngologist. The surgery performed and the medication prescribed is according to standard of care in patients with CRSwNP. Written informed consent is obtained from every participant. The patient is free to withdraw from the study at any time. Collected clinical data will be anonymised with unique patient identification codes.

Adverse events (AEs) to be reported are complications after SPT, ESS and adverse effects from any drug treatment started for CRSwNP. All study-specific AEs reported spontaneously by the participant or observed by the investigators will be recorded each visit. At every follow-up visit or interim telephone contact the investigators should inquire about AEs by asking the patients and by actively screening the patient’s medical file. Serious adverse events (SAEs) possibly related to the study procedure will be reported to the principal investigator within 24 h. The local investigator informs the study coordinator in the Academic Medical Centre and is responsible for reporting SAEs annually to the accredited Medical Ethics Committee that approved the protocol in a line-listing format combined with the annual progress report.

In case of life-threatening SAEs or death, reporting to the accredited Medical Ethics Committee will occur not later than 7 days after the study coordinator’s knowledge of the event.

### Dissemination

Presentations at (inter)national scientific conferences will be part of dissemination. Results will also be published in scientific journals. The raw trial data will be made available to the members of the Dutch Society of Otorhinolaryngology and Head and Neck Surgery.

## Discussion

The timing and indications for ESS in the management of CRSwNP/CRSsNP are mainly based on practitioners’ knowledge. National and international clinical guidelines advise to start with drug treatment for at least 1 month before considering surgical treatment. Based on clinical findings patients start a drug treatment consisting of nasal corticosteroids, eventually supplemented with a short course of systemic corticosteroids or a longer course of antibiotics [3, 5, 6, 26–28]. Patients failing drug treatment are offered a choice between more intensive drug treatment and surgery in addition to drug treatment. Shared decision-making between the otorhinolaryngologist and the patient decides the moment that surgery is needed for relief of symptoms. Because of the chronic nature of the (mucosal) disease, the optimal treatment would be local treatment with medication combined with surgery.

Rudmik et al. have already shown in a Markov decision-tree economic evaluation that ESS would be the most cost-effective intervention compared to continued medical therapy from a long-term perspective, at least with 74% certainty [29]. Limitation of this study, however, is that this economic evaluation was not performed alongside a RCT. The current study will be the first high-quality multicentre RCT (*N* = 238) to evaluate the role of ESS and to assess the cost-effectiveness of ESS in addition to drug treatment compared to drug treatment alone in adults with CRSwNP.

Currently, this trial is being conducted in 15 hospitals in The Netherlands. To the best knowledge of the investigators no other randomised studies to evaluate the same question are currently being performed. The objective is to demonstrate a higher HRQOL after ESS compared to drug treatment only in the treatment of CRSwNP. The outcome measurements are chosen according to experience in the field. Patient symptoms are thought to be an important parameter because patients seek medical advice in case of symptoms, regardless of the extent of disease visible on nasal imaging or with nasendoscopy. The usage of PROMs in clinical trials is growing and HRQOL is a frequently used clinical endpoint in clinical trials for CRS.

After the two-arm randomisation process, the medical intervention consists of any drug treatment that can be given to patients with CRSwNP in routine medical practice. The drug treatment purposefully is not standardised so as to stay closest to standard care. Also, patients’ need for drug treatment varies with time and extent of disease. Naturally, this design enhances diversity; however, it also enhances generalisation of the results in the real-world situation. Surgical intervention consists of ESS, which is described as any surgery performed regularly by the otorhinolaryngologists in The Netherlands. The extent of the surgery is tailored to the extent of disease. This introduces a performance bias; however, this will also be closest to normal care.

The results of this RCT are intended to create a tailored strategy and selective use of ESS in CRSwNP patients. The results will be generalised to the Dutch situation and implemented in clinical guidelines.

### Trial status

The study is currently in the first phase of patient recruitment and inclusion in 15 Dutch hospitals. Enrolment started on 13 February 2015. The anticipated recruitment completion is Summer 2017.

## Additional files

Additional file 1: Table S1. SPIRIT 2013 Checklist: recommended items to address in a clinical trial protocol and related documents. (DOC 122 kb)
Additional file 2: Table S2. Assessment of current clinical control of chronic rhinosinusitis (CRS) (in the last month), EPOS 2012. (JPG 76 kb)

## Abbreviations

AE: Adverse event
BIA: Budget impact analysis
CONSORT: CONsolidated Standards of Reporting Trials
CRS: Chronic rhinosinusitis
CRSsNP: Chronic rhinosinusitis without nasal polyps
CRSwNP: Chronic rhinosinusitis with nasal polyps
CT: Computed tomography
EPOS: European Position Paper on Chronic Rhinosinusitis
ESS: Endoscopic sinus surgery
GCP: Good Clinical Practice
HRQOL: Health-related Quality of Life
iMTA: Institute for Medical Technology Assessment
MEC: Medical Ethics Committee
MLMES: Modified Lund-Mackay Postoperative Endoscopy Score
PNIF: Peak nasal inspiratory flow
PROM: Patient-reported measure of outcome
QALY: Quality-adjusted Life Year
RCT: Randomised controlled trial
SNOT-22: Sinonasal Outcome Test 22
SPT: Skin Prick Test
VAS: Visual Analogue Scale
ZonMw: Dutch Organisation for Health Research and Development
