# Supplementary figures and images for: Endoscopic sinus surgery in adult patients with chronic rhinosinusitis with nasal polyps (PolypESS): study protocol for a randomised controlled trial
Source: Trials. 2017 Jan 23;18:39. doi: 10.1186/s13063-016-1728-z (PMC5259992; doi:10.1186/s13063-016-1728-z)

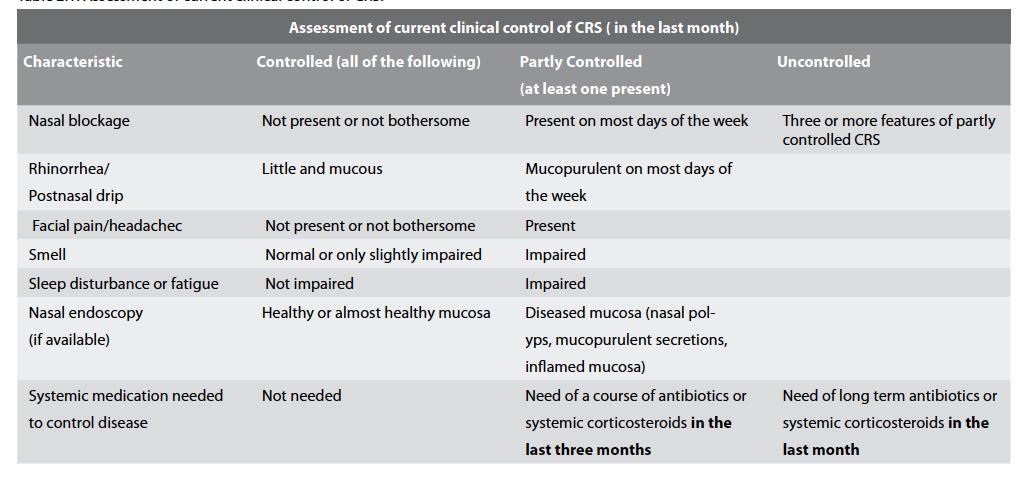

Supplement: Additional file 2: Table S2. — Assessment of current clinical control of chronic rhinosinusitis (CRS) (in the last month), EPOS 2012. (JPG 76 kb) [file 13063_2016_1728_MOESM2_ESM.jpg]
